# Improved Tribological Performance of Amorphous Carbon (a-C) Coating by ZrO_2_ Nanoparticles

**DOI:** 10.3390/ma9100795

**Published:** 2016-09-22

**Authors:** Jinzhu Tang, Qi Ding, Songwei Zhang, Guizhi Wu, Litian Hu

**Affiliations:** 1 State Key Laboratory of Solid Lubrication, Lanzhou Institute of Chemical Physics, Chinese Academy of Sciences, Lanzhou 730000, China; tangjinzhu11@163.com (J.T.); zhangsw@licp.cas.cn (S.Z.); gzwu@licp.cas.cn (G.W.); 2 University of Chinese Academy of Sciences, Beijing 100049, China; 3 Qingdao Center of Resource Chemistry and New Materials, Qingdao 266100, China

**Keywords:** amorphous carbon coating, ZrO_2_ nanoparticles, friction coefficient, wear, polishing effect

## Abstract

Nanomaterials, such as Graphene, h-BN nanoparticles and MoS_2_ nanotubes, have shown their ability in improving the tribological performance of amorphous carbon (a-C) coatings. In the current study, the effectiveness of ZrO_2_ nanoparticles (ZrO_2_-NPs) in lubricating the self-mated nonhydrogenated a-C contacts was investigated in boundary lubrication regime. The results showed that 13% less friction and 50% less wear compared to the base oil were achieved by employing ZrO_2_-NPs in the base oil in self-mated a-C contacts. Via analyzing the ZrO_2_-NPs and the worn a-C surface after tests, it was found that the improved lubrication by ZrO_2_-NPs was based on “polishing effects”, which is a new phenomenon observed between a-C and nanoparticles. Under the “polishing effect”, micro-plateaus with extremely smooth surface and uniform height were produced on the analyzed a-C surface. The resulting topography of the a-C coating is suitable for ZrO_2_-NPs to act as nano-bearings between rubbing surfaces. Especially, the ZrO_2_-NPs exhibited excellent mechanical and chemical stability, even under the severe service condition, suggesting that the combination of nonhydrogenated a-C coating with ZrO_2_-NPs is an effective, long lasting and environment-friendly lubrication solution.

## 1. Introduction

Nowadays, amorphous carbon (a-C) coatings, such as diamond-like carbon (DLC) coatings, have been widely applied in moving mechanical assemblies to improve the energy efficiency and avoid unwanted material wear [1,2,3]. To further enhance the performance of a-C coatings in oil lubricated condition, investigations into the synergetic lubrication between a-Cs and lubricating oils or additives have been extensively conducted for the past decades. However, it was found that most of the successful oil additives (such as ZDDP and MoDTC) that were tailored for metallic surface were less effective for a-Cs due to low reactivity of a-C with those chemically based additives [4,5,6,7,8,9,10].

Recently, researches regarding nanoparticles used as lubricating additive for a-C coatings have been extensively conducted, and the results showed that nanoparticles could significantly improve the tribological performances of a-C contacts [11,12,13,14,15,16,17,18,19]. In traditional metallic friction pairs, the protective tribofilm formed by deposited nanoparticles is the essential tribological mechanism for most of the existing nano-additives (such as Ag, Cu, MoS_2_, WS_2_ and ZrO_2_) [20,21,22,23,24,25,26,27,28]. However, for a-C surfaces that have low adhesiveness and high hardness, the lubrication of nanoparticles may rely on other mechanisms.

Kalin et al. [13] reported that the multi-wall MoS_2_ nanotubes could reduce the friction in a-C:H contacts by up to 50%, and the improved performance was attributed to the ultra-thin MoS_2_ film formed by the exfoliated MoS_2_ nanosheets which strongly adhered on the a-C surfaces. A similar exfoliation-adhesion phenomenon was also observed with carbon nanotubes by Zhang et al. [18,19]. It is inferred that the high adhesion of exfoliated 2D nanosheets lies in the large van der Waals force and low bend stiffness. Meanwhile, the a-C coating, as a smooth and stiff substrate, assists the sliding between the absorbed nanosheets, resulting in a more effective friction reduction than that in steel contacts [13]. However, Zhang et al. [18] also suggested that the exfoliation of nanotubes only occur under high applied load, while under lower load, the friction reduction depended on the rolling of nanotubes. Zeng et al. observed ultralow friction in a-C/Si_3_N_4_ contacts with the aids of h-BN nanoparticles [15]. In their opinion, the reduced friction lies in the nano-bearing effect and the internal sliding between h-BN lattice planes. Berman et al. [29] obtained ultra-low friction in a-C:H/SiO_2_ contacts with the nanoscrolls formed by diamond nanoparticles and graphene. The authors proposed that the nanoscrolls, as nano bearings, reduced contact area and constructed an incommensurate contact with a-C surface. In summary, in a-C contacts, the performances of nanoparticles mainly rely on the rolling motion and structural transformation (such as exfoliation and shearing) of nanomaterials.

However, the exfoliation of nanotubes or shearing of nanoparticles is a depletion process of additives, causing degradation in performance on long-term use. On the other hand, as an important lubrication mechanism of nano-additives, the nano-bearing effect that is based on the rolling of nanoparticles has not been received much attention, partly due to the adhesive nature of steel surfaces that are adverse to the rolling motion of nanoparticles. Recently, Alazami et al. [30] reported significant tribological improvement in steel contacts based on the nano-bearing effect of perfectly spherical carbon with ultra smooth surface.

Theoretically, due to high hardness and non-adhesiveness of a-C surface, it is easier for nanoparticles to realize the nano-bearing effect in a-C contacts. Among nanoparticles, ceramic nanoparticles are very suitable to act as nano-bearings owing to their excellent mechanical, chemical and thermal stabilities, and low toxicity and cost [31]. Therefore, the combination of a-C coating with ceramic nanoparticles should be an effective, long lasting, and environment-friendly lubrication solution for automobile engines, refrigeration compressor and rolling bearings. However, to the best of our knowledge, there is no literature focused on the subject.

In this paper, the zirconia nanoparticles (ZrO_2_-NPs), which exhibit extremely low adhesive interaction with a-C material (reported by Lu et al. [32]), were selected, and the tribological effects of ZrO_2_-NPs in a-C contacts were investigated. Quite promising results were obtained that both the friction and wear in a-C contacts were reduced by ZrO_2_-NPs. The “polishing effect”, a new phenomenon observed between a-C and nanoparticles, was reported, which is the critical mechanism behind the improved tribological performance.

## 2. Experimental Section

### 2.1. Materials and Lubricants

**Figure 1:**
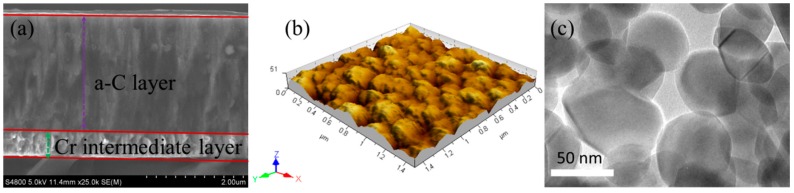
(**a**) The cross-sectional morphology; and (**b**) the surface topography of as-deposited a-C coating; and (**c**) the TEM micrographs of used ZrO_2_-NPs.

The disks for the experiments were made of GCr15 (AISI52100) steel having a hardness of 8.3 GPa after heat treatment and a roughness Ra lower than 0.02 μm. The steel balls were commercially available, standard bearing steel (AISI52100) balls with the diameter of 6 mm. Some of the disks and balls were directly used as self-mated steel contacts in the tribo tests, while others were coated with nonhydrogenated a-C on the UDP650 magnetron sputtering deposition system (Teer Coatings Ltd., Hartlebury, UK). Table 1 lists the process parameters for coating deposition. Figure 1a,b shows the cross-sectional morphology and surface topography of as-deposited a-C coating. The total thickness of as-deposited a-C coatings is 3 μm with the roughness Ra of 5.9 nm.

Commercially available ZrO_2_-NPs (99.99%, 50 nm, Aladdin Industrial Corporation, Shanghai, China) were selected as lubricating additives. Figure 1c shows the HRTEM images of ZrO_2_-NPs, showing the diameter of ZrO_2_-NPs is in the range of 30–60 nm. According to the well-established Hall–Petch (H–P) relationship, the hardness of ZrO_2_-NPs is higher than the bulk hardness of 6.5 on Mohs scale (equivalent to 9.8 GPa), even taking into account the inverse H–P relation [22]. The used base oil was the additive-free poly-alpha-olefin (PAO4) oil having the viscosity of 16.8 mm^2^/s and pressure–viscosity coefficient of 17.08 GPa^−1^ at 40 °C. The ZrO_2_-NPs were physically dispersed in the PAO oil at the concentrations of 1 wt % using an ultrasonic bath for 30 min before each tribo test. No surfactants or dispersant agents were added in order to eliminate their influence in the experiment results. The ZrO_2_-NPs dispersion could maintain stablity for 1 h as presented in the Supplementary Materials.

### 2.2. Tribological Evaluation

**Figure 2:**
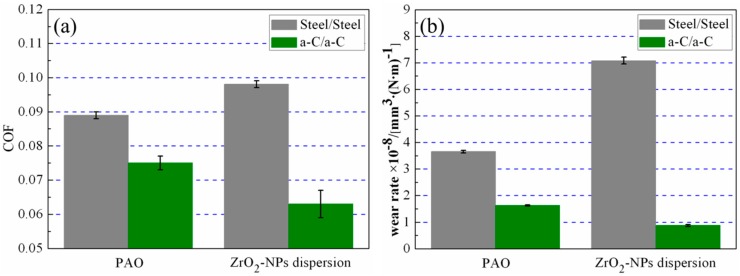
(**a**) The average COFs; and (**b**) wear rates of steel/steel and a-C/a-C contacts under the lubrication of pure PAO and ZrO_2_-NPs dispersion.

Tribological evaluations, in which self-mated steel and a-C contacts were used, were performed on a tribometer (UMT-2, CETR, Campbell, CA, USA) with a ball-on-disk configuration. The applied load was 20 N resulting in an initial Hertzian contact stress about 1.1 GPa. The upper ball slides against the disk at the frequency of 5 Hz with the amplitude of 5 mm for 30 min. The same amount of lubricants (0.2 mL) was spread on the disk before each test. All experiments were repeated 3–4 times to ensure statistically relevant results and the average values are presented in the Figure 2.

The theoretical minimum film thickness (*h_min_*) and dimensionless lambda (λ) ratio were calculated using the Equations (1) and (2), respectively [33].

(1) $$\frac{h_{min}}{R^{\prime }}\;=3.63{\left(\frac{U\eta_{0}}{E^{\prime }R^{\prime }}\right)}^{0.68}{\left(\alpha {Ε}^{\prime }\right)}^{0.49}{\left(\frac{W}{E^{\prime }R^{\prime 2}}\right)}^{-0.073}\left(1-e^{-0.68k}\right),$$

(2) $$\lambda =\frac{h_{min}}{\sqrt{{R_{q1}}^{2}+{R_{q2}}^{2}}},$$

where *R′* is the reduced radius of curvature, *U* is the entraining surface velocity, *W* is the normal load, *E′* is the reduced Young’s modulus, *η*_0_ is the dynamic viscosity, *α* is the pressure–viscosity coefficient, *R_q_*_1_ is the surface roughness of ball, and *R_q_*_2_ is the surface roughness of disc. Under the test condition, the calculated *λ* is 0.001, suggesting that the lubrication state of friction pairs is in boundary regime (*λ* < 1).

### 2.3. Surface Analysis

After the tribo tests, the morphology, distribution and chemical composition of ZrO_2_-NPs on the worn a-C surface were analyzed with the Field Emission Scanning Electron Microscope (FESEM) (JSM-6701F, JEOL, Tokyo, Japan) and X-ray Photoelectron Spectroscopy (XPS) (PHI-5702, Physical Electronics, Al–Kα irradiation at 29.4 eV, Chanhassen, MN, USA), respectively. Before the analysis, the worn a-C surfaces were gently rinsed with acetone to remove the lubricating oil, and special care was taken to preserve the residual ZrO_2_-NPs intact in the wear tracks. After analyses, the residual ZrO_2_-NPs were thoroughly removed by washing with acetone in ultrasonic bath for the following characterizations.

The surface profile of the wear tracks on disks was measured with an optical 3D profiler (MicroXAM, ADE Phase-Shift, Tuscon, AZ, USA). The specific wear-rate coefficient *K* was calculated using the equation by Archard and Hirst: *K* = *V*/(*FS*), where *V* is the wear volume, *F* is the applied load and *S* is the total sliding distance [34]. The micro topography of worn a-C surfaces was analyzed via Atomic Force Microscope (AFM) (AFM-5500, Agilent Technologies, Chandler, AZ, USA). Images were taken in the tapping mode, using the commercially available type II MAC levers (nominal force constant: 2.8 N/m) at a driving frequency of 75 KHz and a non-conductive silicon nitride tip (tip size: 10 nm).

Raman spectroscopy (Lab JY-HR800, Horiba, λ: 532 nm, Kyoto, Japan) and XPS spectroscopy were employed to evaluate the structural changes in a-C surface after tribological tests. The mechanical properties of a-C coating after tribo tests were investigated with a nano-indenter (Nanoindentation Tester, CSM Instruments, Peseux, Switzerland, indentation depth: 100 nm). The indentation hardness (H) and elastic modulus (E) were calculated with the Oliver–Pharr model from simulated P–h curves (Poisson’s ratio: 0.3).

## 3. Results and Discussion

### 3.1. Tribological Results

Figure 2a,b presents the coefficients of friction (COF) and the wear rates of steel/steel and a-C/a-C contacts, respectively. In steel contacts, compared with pure PAO lubricated condition, the ZrO_2_-NPs remarkably increase the friction and wear. However, in a-C contacts, the adding of ZrO_2_-NPs in PAO is very effective in friction and wear reduction. As shown in Figure 2, the COFs of a-C contacts were reduced by 13%, and the wear rates were lowered by 50%.

**Figure 3:**
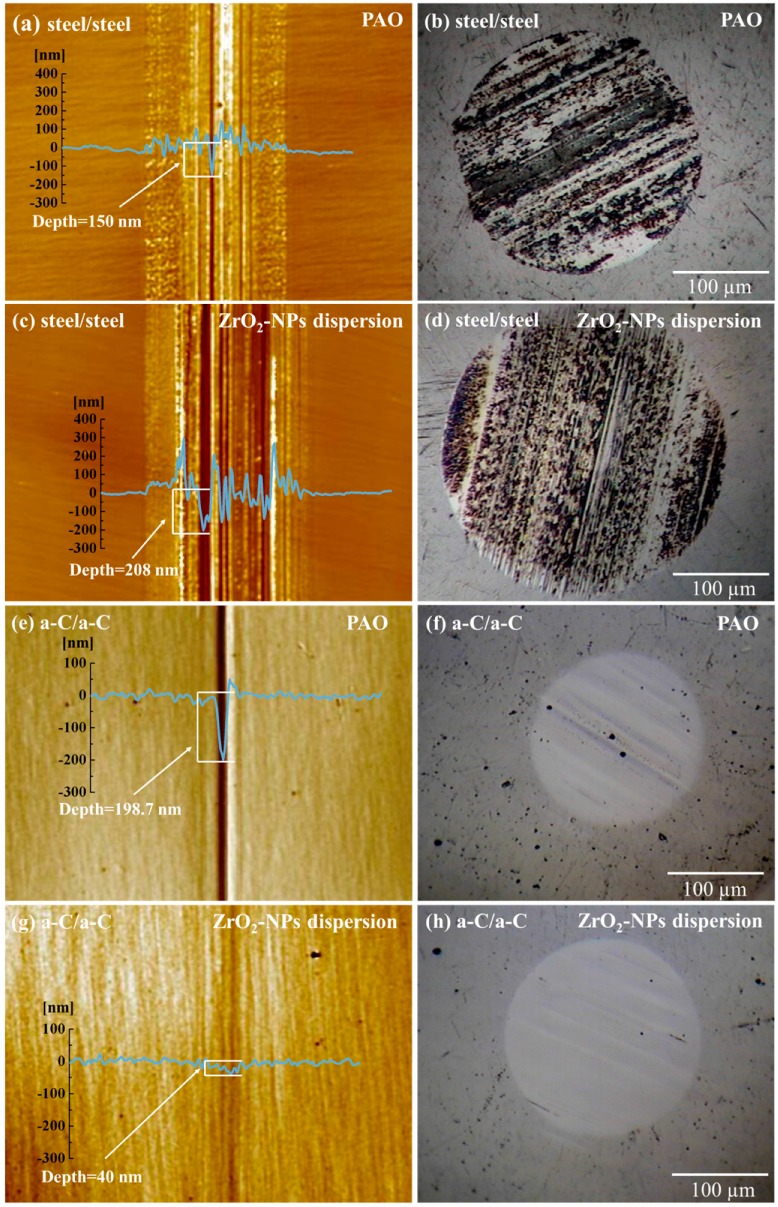
The morphology and cross-sectional profile of the wear tracks on: (**a**,**c**) steel; and (**e**,**g**) a-C disks. The optical micrographs of the wear scar on: (**b**,**d**) steel balls; and (**f**,**h**) a-C coated balls.

Figure 3 shows the morphology and cross-sectional profile of the wear tracks on the a-C and steel disks, and corresponding wear scar on the slider balls. It shows that, in pristine PAO, the steel contacts exhibit typical adhesive wear indicated by the obvious large protruding peaks in the wear tracks region (Figure 3a). Lubricated with ZrO_2_-NPs dispersion, deep grooves could be observed on the wear track and wear scar of steel contacts, and the diameter of wear scar on steel ball is increased by ZrO_2_-NPs (Figure 3d), suggesting that the ZrO_2_-NPs, acting as abrasive particles, plowed the steel surfaces during friction.

In a-C contacts, quite different effects were observed. Under the lubrication of pristine PAO oil, abrasive wear took place in a-C contacts, producing deep grooves on the surfaces of a-C disk and a-C coated slider ball (Figure 3e,f). Introducing ZrO_2_-NPs into PAO oil could remarkably alleviate the abrasive wear, which is evidenced by the shallower grooves on the wear track and the smooth surface of wear scar (Figure 3g,h). By comparing the tribological effects of ZrO_2_-NPs in steel and a-C contacts, it could be concluded that the ZrO_2_-NPs exhibit remarkable synergetic lubricating effects with analyzed nonhydrogenated a-C coating, and the following section will focus on the synergetic effect between them, and elucidating its mechanisms.

### 3.2. The Synergetic Lubricating Mechanism between a-C and ZrO_2_-NPs

#### 3.2.1. The Characterizations of the Residual ZrO_2_-NPs on the Worn a-C Surface

**Figure 4:**
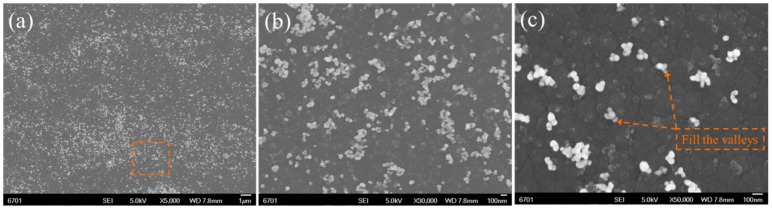
(**a**) FESEM images of the residual ZrO_2_-NPs on the worn a-C surfaces after friction; (**b**) magnified details of the region marked in Figure 4a; and (**c**) the distribution state of ZrO_2_-NPs.

Through the pretreatment methods as stated in Section 2.3, it is possible to investigate the morphology and distribution of the residual ZrO_2_-NPs on the worn a-C surfaces after tribo tests, which is helpful to understand the role of ZrO_2_-NPs in advancing the tribological performances of a-C contacts. Figure 4 shows the FESEM images of the residual ZrO_2_-NPs on the wear track on a-C disk. The ZrO_2_-NPs are evenly distributed on the worn track (Figure 4a), and most of the ZrO_2_-NPs maintain their initial spherical morphology after being tested under boundary lubrication condition (Figure 4b). The distribution of ZrO_2_-NPs (in Figure 4c) shows that the ZrO_2_-NPs are preferentially distributed at the boundaries of micro-regions on a-C surfaces. Referring to the topography of as-deposited a-C coating in Figure 1b, the micro-regions correspond to the micro-bumps on a-C and the boundaries are the valleys between them. Thus, it reveals that the ZrO_2_-NPs could fill the nano-scale valleys on a-C surfaces.

**Figure 5:**
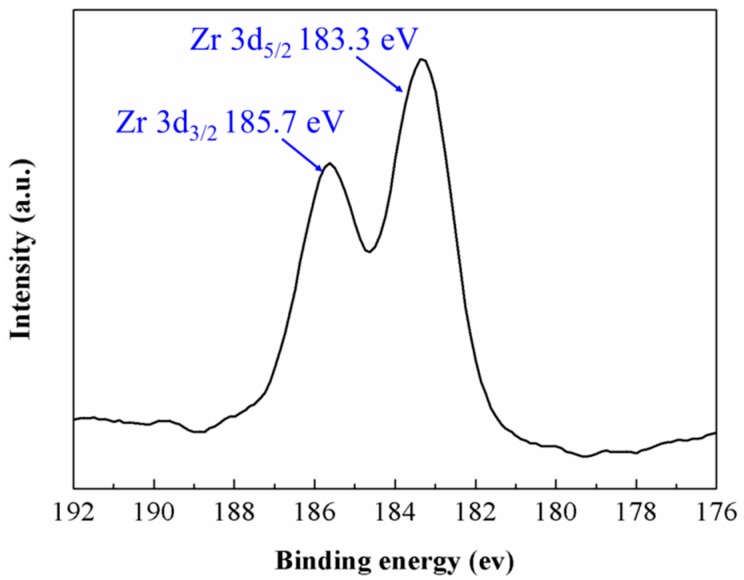
The XPS spectra of the residual ZrO_2_-NPs on the worn a-C surface.

Not only the morphology but also the compositional properties of ZrO_2_-NPs were preserved during tribo tests. In Figure 5, the XPS spectra of the residual ZrO_2_-NPs exhibit two spin–orbit components, the 3d_5/2_ at 183.3 eV and 3d_3/2_ at 185.7 eV, which correspond to the Zr^4+^ in oxidation state [35,36]. Thus, no chemical reaction occurred between ZrO_2_-NPs and a-C coating during friction, indicating that the improved tribological performance of a-C contacts by ZrO_2_-NPs should be ascribed to physically based effects such as the nano-bearing or valleys-filling effects. To further confirm the above deduction, worn a-C surfaces were analyzed in detail after the residual ZrO_2_-NPs were thoroughly removed.

#### 3.2.2. The Characterizations of the Worn a-C Surfaces

**Figure 6:**
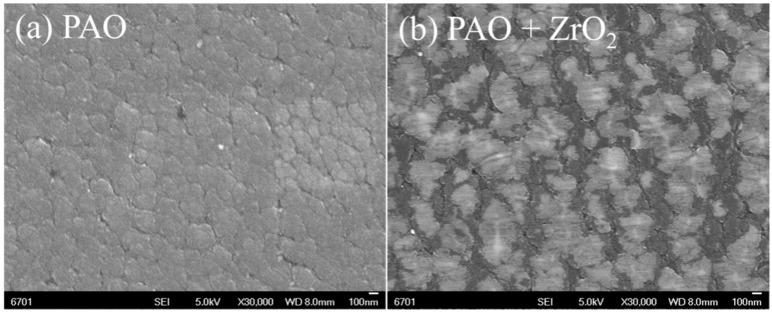
The FESEM images of worn a-C surfaces under the lubrication of: (**a**) pristine PAO oil; and (**b**) ZrO_2_-NPs dispersion.

Figure 6 presents the FESEM images of worn a-C surfaces after tribo tests. With pure PAO oil, the worn a-C surface shows compacted micro-regions with the diameter range around 100~300 nm. In comparison, with ZrO_2_-NPs dispersion, a special damage appearance was produced. As shown in Figure 6b, numerous white spots can be found on the worn a-C surface, and the white spots are located on the center of the micro-regions.

**Figure 7:**
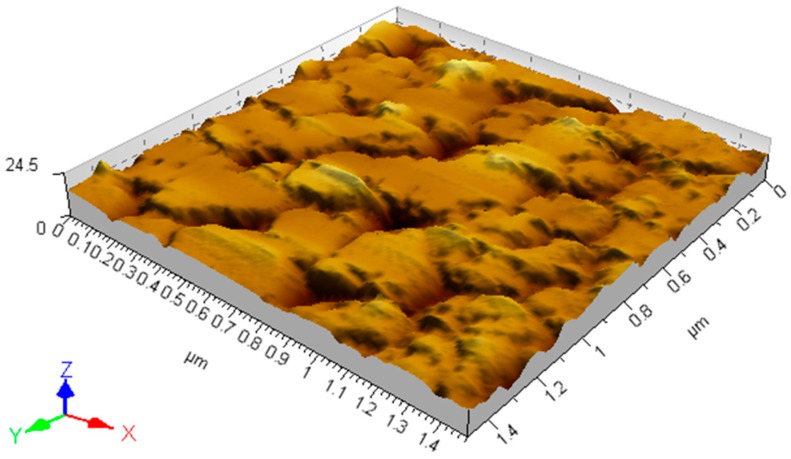
The topography of worn a-C surface under the lubrication of ZrO_2_-NPs dispersion.

The topography of the white spot in Figure 6b was analyzed via AFM. Results show that the white spots have the plateau-like topography, and the sliding traces of ZrO_2_-NPs can be observed on the top surfaces of plateaus (Figure 7). Clearly, the resulted plateau-like topography on analyzed a-C surface should be caused by the polishing from the ZrO_2_-NPs. It is noteworthy that such “polishing effect” could only be observed with ZrO_2_-NPs in current study. As presented in the Supplementary Materials, when we investigated the tribological effects of W nanoparticles that contain particles with submicron and micron size (Figure S1), micro-scratches, instead of micro-plateaus, were produced on the analyzed a-C surface (Figures S2 and S3), suggesting that the “polishing effect” between a-C and the third-body particles is highly dependent on size and size distribution of the particles.

**Figure 8:**
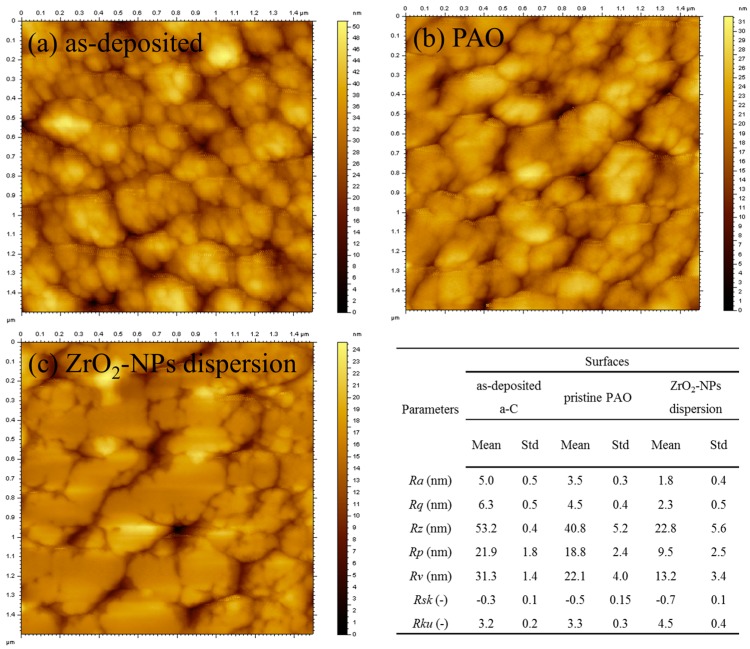
The topography and roughness parameters of: (**a**) as-deposited a-C coating; and the worn a-C surfaces under the lubrication of: (**b**) pure PAO; and (**c**) ZrO_2_-NPs dispersion.

Figure 8 presents the topography and corresponding roughness parameters of as-deposited a-C coating and the worn a-C surface under the lubrication of pure PAO and ZrO_2_-NPs dispersion. As shown in Figure 8c, under the “polishing effect” of ZrO_2_-NPs, the micro-bumps on a-C surface transformed into micro-plateaus with significantly decreased *Ra* and *Rq*. The *Rz* is decreased to 23 nm, which is smaller than the diameter of ZrO_2_-NPs. In addition, the *Rsk* (Skewness of the profile height distribution) becomes more negative, indicating that the asperities were preferentially removed during polishing process. The *Rku* (Kurtosis of the profile height distribution) increases from 3.2 to 4.5, meaning that the height distribution become narrow, i.e., the height of micro-plateaus is very uniform. In comparison, under the lubrication of pure PAO (Figure 8b), although the *Ra*, *Rq*, *Rz* and *Rsk* decrease to some extent, the *Rku* only changed a little and is very close to 3, indicating that, under the lubrication of pure PAO, the profile height distribution of the worn a-C surface is in Gaussian distribution, which is similar to the as-deposited a-C coating.

**Figure 9:**
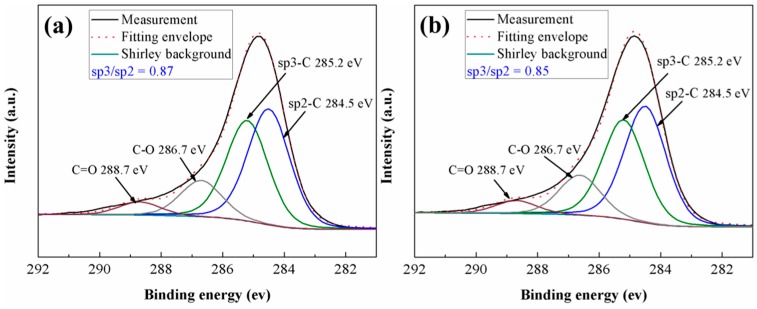
The fitting results of the C1s peak in XPS spectra of: (**a**) as-deposited a-C; and (**b**) the worn a-C surface under the lubrication of ZrO_2_-NPs dispersion.

The XPS were employed to investigate the changes of surface chemical composition of a-C during friction. Figure 9 shows the C1s peak in the XPS spectra obtained from the as-deposited and the worn a-C surface that was lubricated with ZrO_2_-NPs dispersion. The C1s peaks were fitted by (80% Gaussian + 20% Lorentzian) function. The full width at half maximum (FWHM) of the fitted peaks was set at 1.60 eV, and four components centered at 284.5 eV (Csp2), 285.2 eV (Csp3), 286.7 eV (C–O), and 288.7 eV (C=O) were obtained according to the references [37,38,39]. The areas under the Csp2 and Csp3 peaks were used to determine the Csp3/Csp2 ratio in a-C. As shown in Figure 9, compared with as-deposited a-C, the Csp3/Csp2 ratio of worn a-C surface shows no obvious changes after tribo tests, indicating that the ZrO_2_-NPs did not affect the surface chemical composition of a-C during friction.

**Figure 10:**
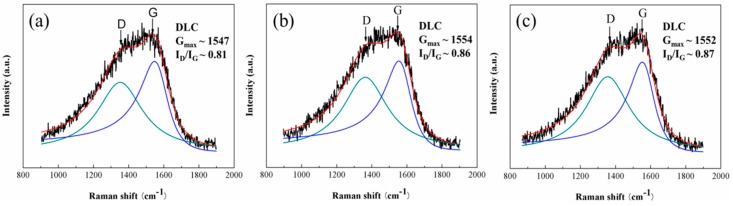
The Raman spectra of: (**a**) as-deposited a-C; and the wear tracks on a-C under the lubrication of: (**b**) pure PAO; and (**c**) ZrO_2_-NPs dispersion. The insets show the fitting results of G peak position (G_max_) and the I_D_/I_G_ ratio (height ratio).

Then, the bonding structure, especially the configuration of sp2 hybridized carbon in a-C, were investigated using Raman spectroscopy. Figure 10 shows the Raman spectra obtained from the as-deposited a-C and the worn a-C surface. All spectra show the similar broad peak between 1100 and 1750 cm^−1^, which is typical for a-C, suggesting the a-C coating preserved the amorphous structure during friction. The spectra were deconvoluted by the BWF + Lorentzian pair into two main Raman bands, one located at 1550 cm^−1^ (G band), and the other at 1360 cm^−1^ (D band). The program FITYK was applied for peak fitting using the Levenberg–Marquard algorithm for nonlinear least-square optimization. The fitting results of G peak position (G_max_) and the I_D_/I_G_ ratio (peak height ratio) from the Raman spectra are inserted in Figure 10.

The fitting results show that the G peaks shift to higher frequencies with increased I_D_/I_G_ ratios after being tested in both lubricants. According to the “Three Stage Model” proposed by Ferrari et al. [40], the microstructure of analyzed a-C coating belong to the Stage 2, in which the G peak position is related with the ordering of bond-angle and bond-bending of Csp2 in rings, while I_D_/I_G_ ratios is dependent on the number of ordered aromatic rings. Therefore, after the tribo tests, the blue shift of G_max_ with the increased I_D_/I_G_ ratio indicate that the Csp2 in a-C surface became more ordered during friction.

During friction, the ordering of Csp2 in a-C was triggered by the frictional heat, and the high contact stress exerted by the abrasive particles could promote the transformation [41]. However, as suggested by the fitting results, the ZrO_2_-NPs did not promote the transformation during friction, possibly due to the nano-scale size of the ZrO_2_-NPs, which is even smaller than the asperities on the analyzed a-C coating, and the uniform size distribution of ZrO_2_-NPs, which ensure the load is evenly supported by the nanoparticles in the contact region.

**Figure 11:**
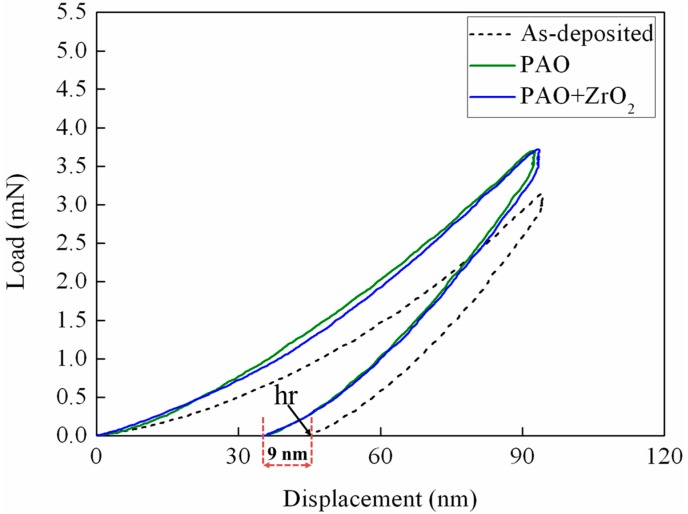
The load−displacement curves obtained from the as-deposited a-C and from the wear tracks on a-C under the lubrication of pure PAO and ZrO_2_-NPs dispersion. The inset shows the average results of hardness (H) and elastic modulus (E) from four parallel tests (Figure S5), hr: residual depth after completed unloading.

Via nano-indentation tests, it is found that the worn a-C surfaces under the lubrication of ZrO_2_-NPs dispersion and pure PAO exhibit similar indentation-recovery behavior (Figure 11). The extracted coating hardness (H) and elastic modulus (E), which were obtained by averaging the results from four parallel tests (see more details in Supplementary Materials), are similar for two test conditions (Figure 11, inset). Additionally, it is interesting to note that the H and E obtained from the worn a-C surfaces are slightly higher than those obtained from the as-deposited a-C.

The phenomenon should be related with the non-uniform cross-sectional structure of a-C coating. As reported by Ferrari et al., the magnetron sputtered a-C coating (thickness: 360 nm) consists of a dense bulk layer (1.7 g/cm^3^) and a less dense surface layer (1.15 g/cm^3^, thickness: 5 nm), which is originated from the subplantation mechanism during deposition [42]. The difference in hr (9 nm) between as-deposited a-C and the worn a-C in Figure 11 implies the existence of the surface layer in analyzed a-C coating. In addition, a high coating hardness is necessary to realize the beneficial effects of ZrO_2_-NPs. As presented in the Supplementary Materials, for the Cr-doped a-C with the hardness of 9.9 GPa, the ZrO_2_-NPs dispersion cannot reduce the friction, but can significantly increase the wear rate. Thus, the ZrO_2_-NPs, which have hardness higher than 10 GPa, may be not suitable for the a-C coatings with the hardness below 10 GPa.

### 3.3. The Tribological Mechanism of ZrO_2_-NPs in a-C Contacts

As presented above, the ZrO_2_-NPs exhibit adverse tribological effects in steel contacts, but effectively reduce the friction and wear in a-C contacts. Due to the “polishing effect” of ZrO_2_-NPs during friction, a special topography was obtained on a-C surface with no influences in the compositional and mechanical properties of analyzed a-C coating. However, the topography of contact surfaces will significantly affect the distribution and sliding/rolling of the nanoparticles between them. Actually, the bumpy surface of as-deposited a-C coating is not favorable for the rolling of ZrO_2_-NPs. However, after polished by ZrO_2_-NPs, the micro-bumps transformed into micro-plateaus with extremely smooth surface and uniform height, which is suitable for ZrO_2_-NPs to act as nano-bearings to reduce the friction and wear.

**Figure 12:**
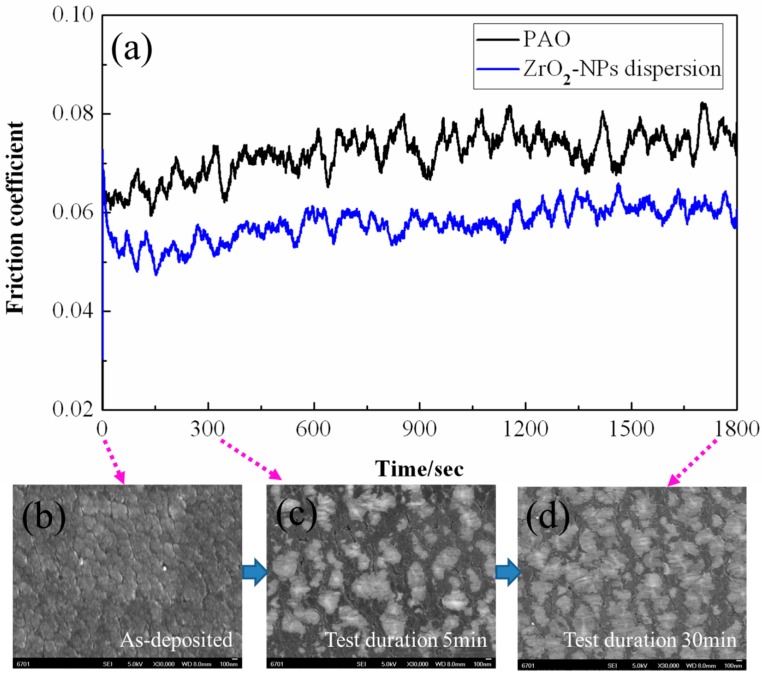
(**a**) The variation of COF of a-C contacts with respect to sliding time. The FESEM images of: (**b**) the as-deposited a-C surface; and the worn a-C surface under the lubrication of ZrO_2_-NPs dispersion for: (**c**) 5 min; and (**d**) 30 min.

By specially focusing on the morphology evolution of a-C surface under the lubrication of ZrO_2_-NPs dispersion, it is found that the a-C surface has been finely polished by ZrO_2_-NPs within 5 min (1500 cycles) after the sliding started (Figure 12c). The reason why the a-C surface could be polished in such a short time may be because the analyzed a-C coating contains a less dense surface layer, as has been discussed above.

**Figure 13:**
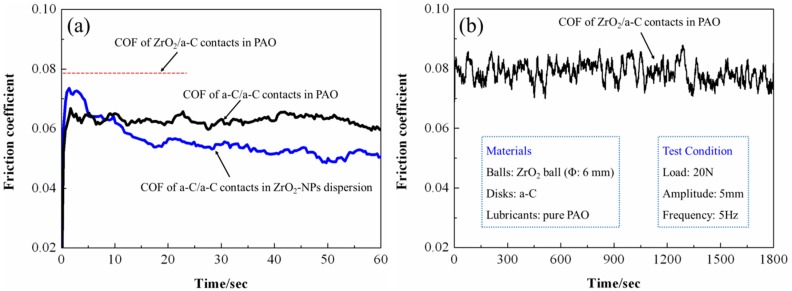
(**a**) The COF variation of a-C contacts during the initial stage under the lubrication of pure PAO and ZrO_2_-NPs dispersion; (**b**) The COF variation of ZrO_2_/a-C contacts under the lubrication of pure PAO, the inset presents the test conditions.

Figure 13a shows the COF variation of a-C contacts during the first 1 min of tribo tests. In the initial stage, the COF of ZrO_2_-NPs lubricated a-C contacts is higher the COF of pure PAO lubricated condition, but is close to the COF of PAO lubricated ZrO_2_/a-C contacts under the same test condition (Figure 13b). It is speculated that the high initial COF of ZrO_2_-NPs dispersion should be related with the “polishing” process, during which most of the ZrO_2_-NPs are sliding on the a-C surface. At the initial running-in stage, the ZrO_2_-NPs could not roll freely on the bumpy a-C surface. Under the shearing action, the motion of ZrO_2_-NPs is blocked by the micro-bumps due to their similar dimension. The ZrO_2_-NPs will exert a high stress on the micro-bumps, and then polish the surface of the micro-bumps. This also explains the reason why the “polishing effect” on analyzed a-C coatings was observed with ZrO_2_-NPs, and could not be observed with the particles of micron size (see Supplementary Materials).

Once the a-C surface was polished and the micro-plateaus were formed, the ZrO_2_-NPs between sliding surfaces should be in rolling state, because the siding of ZrO_2_-NPs could not lower the friction of a-C contacts (COF_ZrO__₂__/a-C_ > COF_a-C/a-C_). However, if the ZrO_2_-NPs are in pure rolling state, the COF should be much lower than the presented results. Therefore, it is inferred that the ZrO_2_-NPs were in a rolling-sliding state with high sliding ratio. Additionally, because the ZrO_2_-NPs can fill the valleys between the micro-plateaus (Figure 4c), the real contact area between contacted a-C surface is increased, which lowers the contact stress and reduces the wear.

Therefore, although the improved tribological performances of a-C coatings by ZrO_2_-NPs can be ascribed to the nano-bearing effect, which lowers the friction, and the valleys-filling effect, which reduces the wear, the “polishing effect” is critical for the acting of these mechanism. It needs to be pointed out that the “polishing effect” between nanoparticles and a-C highly depends on the diameter of nanoparticles and the mechanical properties of a-C coatings. So the selection of nanoparticles should be based on the topography and mechanical properties of used a-C coating. Otherwise, severe scratch damages and high wear will be resulted, as what is presented in the Supplementary Materials and the previous studies [43].

In addition, the “polishing effect” is also dependent on the cross-sectional structure of a-C coatings. Since other kinds of a-Cs, such as ta-C, a-C:H and ta-C:H, have quite different cross-sectional structure (especially the thickness of surface layer) from the analyzed a-C coating in current study [43], whether these a-C coatings can be “polished” by ZrO_2_-NPs is still unknown. Thus, the beneficial effects of ZrO_2_-NPs observed in current study are only restricted to the magnetron sputtered a-C coatings which have a significant surface layer structure and the high bulk hardness above 15 GPa. In the future, it is necessary to conduct a systematical investigation into the tribological effects of ZrO_2_-NPs on other kinds of a-C coatings to elucidate the applicability of ZrO_2_-NPs in a-C friction pairs.

## 4. Conclusions

The addition of ZrO_2_-NPs to PAO oil significantly improved the tribological performance of self-mated a-C contacts in boundary lubrication regime. At the concentration of 1 wt %, the friction of a-C contact pairs was lowered by 13%, and the wear was reduced by 50%. The “polishing effect”, which is the critical mechanism behind the improved tribological performance, was reported. Under the polishing effect of ZrO_2_-NPs, micro-plateaus with extremely smooth surface and uniform height were produced on the analyzed a-C surface. The resulted topography of a-C coating was suitable for ZrO_2_-NPs to act as nano-scale ball bearings to lower the friction and wear by rolling and sliding.

It was found that the “polishing effect” between a-C and nanoparticles highly depends on the diameter of nanoparticles, as well as the mechanical properties and cross-sectional structure of a-C coatings. Thus, the beneficial effects of ZrO_2_-NPs observed in the current study are only restricted to the magnetron sputtered nonhydrogenated a-C coatings that have a significant surface layer and bulk hardness above 15 GPa. In the future, it is necessary to conduct a systematical investigation on other kinds of a-C coatings to elucidate the applicability of ZrO_2_-NPs to be used as lubricating additives for a-C coated friction pairs.

## Figures and Tables

**Table 1:** The process parameters for coating deposition.

Item	Parameters
Coating method	Magnetron sputtering deposition
Carbon source	High-purity graphite
Intermediate layer	Cr
Base pressure	1 × 10^−3^ Pa
Working pressure	Ar, 16 sccm, 0.12 Pa
Bias Voltage	−300 V: Cr intermediate layer
	−70 V: a-C layer
Target current	3.0 A: Cr target
	3.5 A: Graphite target
Deposition time	960 s: Cr intermediate layer
	18,000 s: a-C layer

## Supplementary Materials

The following are available online at www.mdpi.com/1996-1944/9/10/795/s1. Figure S1: FESEM images of the residual W-NPs on the worn a-C surfaces after tribo tests; (b) show the magnified details of the region marked in the Figure S1a; Figure S2: The FESEM images of the worn a-C surfaces when tested with W-NPs. (b,c) show the details of the region marked in Figure S2a; Figure S3: The topography and (b) line profile from the white line of the worn a-C surfaces when tested with W-NPs; (c) The wear rates of a-C lubricated with pristine PAO and PAO with W-NPs and ZrO_2_-NPs; Figure S4: The load−displacement curves in the nano-indentation tests performed on the (a) as-deposited a-C surface and the worn a-C surface when lubricated with (b) pristine PAO oil and (c) PAO with ZrO_2_-NPs, respectively; Figure S5: (a) The nano-indentation results of Cr doped a-C coating; (b) The COF of Cr doped a-C contacts under the lubrication of pure PAO and ZrO_2_-NPs dispersion; (c) The wear rates of a-C and Cr-doped a-C under the lubrication of pure PAO and ZrO_2_-NPs dispersion; Figure S6: Digital images of dispersion of ZrO_2_-NPs in PAO oil. The time for each picture is noted on the respective picture: (a) the as-prepared ZrO_2_-NPs dispersion; and the dispersion after standing for (b) 1 h; (c) 2 h; (d) 3 h and (e) 12 h. Concentration of ZrO_2_-NPs: 1 wt %.

## Abbreviations

The following abbreviations are used in this manuscript:

a-C	Amorphous carbon
DLC	Diamond-like carbon
ZrO_2_-NPs	Nanoparticles of zirconia
Std	Standard deviation
Ra	Arithmetic mean deviation of the profile
Rq	Root mean square deviation of the profile
Rz	Maximum height of the profile
Rp	Maximum profile peak height
Rv	Maximum profile valley depth
Rsk	Skewness of the profile height distribution
Rku	Kurtosis of the profile height distribution
hr	Residual depth after completed unloading
