# Supplementary material for: Improved Tribological Performance of Amorphous Carbon (a-C) Coating by ZrO2 Nanoparticles
Source: Materials (Basel). 2016 Sep 22;9(10):795. doi: 10.3390/ma9100795 (PMC5456616; doi:10.3390/ma9100795)
Supplement: Supplementary file 1 [file materials-09-00795-s001.pdf]

Supplementary Materials: Improved Tribological Performance of Amorphous Carbon (a-C) Coating by ZrO_2 Nanoparticles

Jinzhu Tang, Qi Ding, Songwei Zhang, Guizhi Wu and Litian Hu

Section S1. The Effects of Particles Size on the Wear Behavior of a-C Coating

Preliminary researches were carried out to study the effects of particle size on the wear behavior of a-C coating. The used nanoparticles are W (W-NPs), which have similar bulk hardness with ZrO_2 (W: 7.5, ZrO_2: 6.5, Mohs scale), but contain larger particles with the diameter of submicron and micron scale (Figure S1). The tribo-tests were performed under the same conditions with the ones used for ZrO_2-NPs.

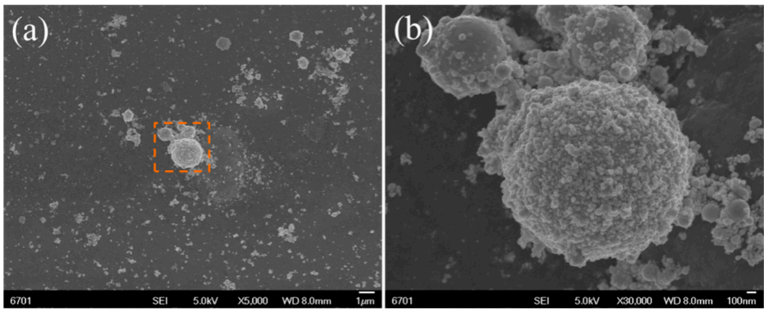

Figure S1. FESEM images of the residual W-NPs on the worn a-C surfaces after tribo-tests; (**b**) show the magnified details of the region marked in the (**a**).

After tested with W-NPs, numerous short scratches with cometary shape along the sliding direction were observed on the worn a-C surface (Figure S2a). The typical morphology correlated with the "polishing effects" (Figure 6b) could not be observed on the worn a-C surface. The magnified images show that severe plastic deformation occurred on a-C surface at the end of scratch (Figures S2b and S3a). The width of scratches were in the range of 1~3.5 μm with a maximum depth of 200 nm (Figure S3b). So the micro-bumps (height < 50 nm) on a-C surface have no influence in the motion of those W particles of micron size.

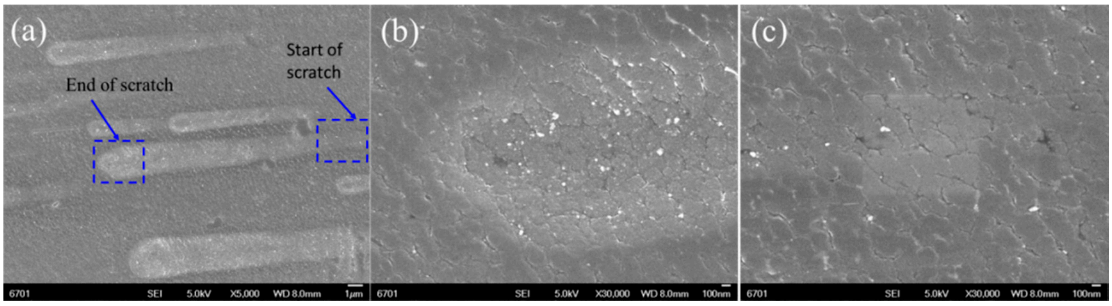

Figure S2. The FESEM images of the worn a-C surfaces when tested with W-NPs. (**b**,**c**) show the details of the region marked in (**a**).

We think the scratches were caused by the indenting and sliding of those larger W particles on a-C surface. The inhomogeneous size distribution of the W particles also increases the stress concentration which intensified the plastic deformation on a-C coating and raised the wear rate (Figure S3c). Therefore, the synergetic lubrication between a-C and hard particles are dependent on the particles' size and their size distribution, which is needed to be systematically studied in the further works.

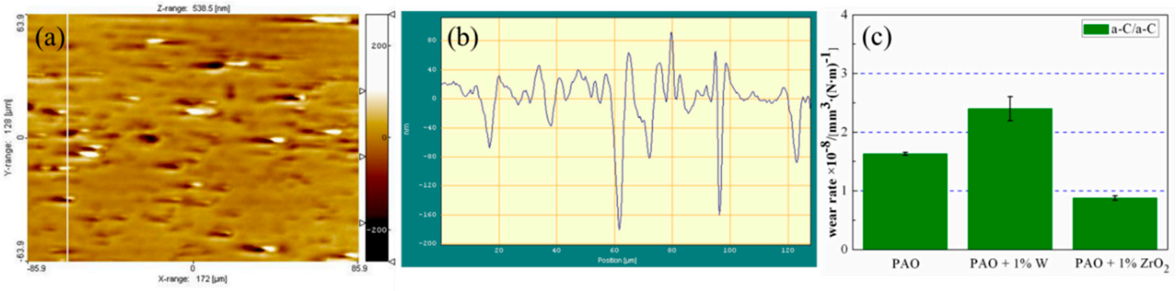

Figure S3. (**a**) The topography and (**b**) line profile from the white line of the worn a-C surfaces when tested with W-NPs; (**c**) The wear rates of a-C lubricated with pristine PAO and PAO with W-NPs and ZrO_2-NPs.

Section S2. Detailed Information for the Mechanical Characterization of the Worn a-C Surface

Figure S4 displayed the load–displacement curves in nano-indentation tests. The tests were performed on the as-deposited a-C coatings and on the wear tracks on a-C after tribo-tests. For each sample, the indentation was performed for at least 4 times to ensure the reliability of the obtained results. As shown in Figure S4, for each sample, the load–displacement curves exhibit high reproducibility, but the data scattering on the worn a-C surface is higher than those obtained on as-deposited a-C, which may be ascribed to the uneven surface of wear tracks.

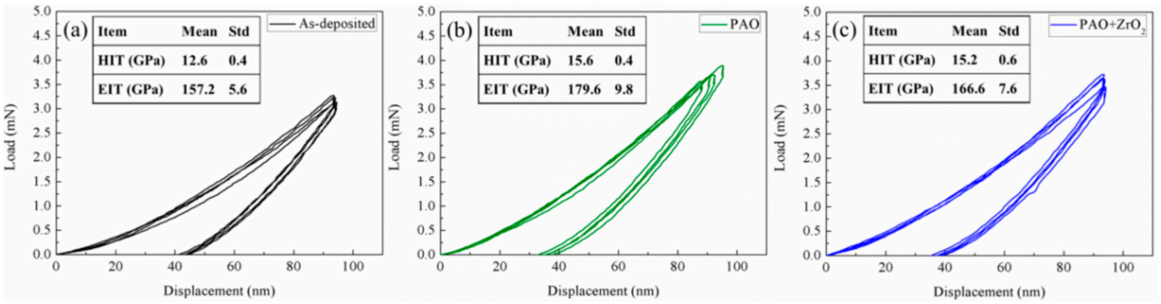

Figure S4. The load–displacement curves in the nano-indentation tests performed on the (**a**) as-deposited a-C surface and the worn a-C surface when lubricated with (**b**) pristine PAO oil and (**c**) PAO with ZrO_2-NPs, respectively.

On the worn a-C surface, the maximum loads at the defined indentation depth are similar for the two lubrication conditions (Figure S4b,c), but are higher than the ones obtained on as-deposited a-C (Figure S4a). The insets in Figure S4 show the mean values of the elastic modulus (E) and the hardness (H) which were calculated from the load–displacement curves. The results suggest that the coating hardness and elastic modulus were raised after friction. The reason was explained in the main text.

Section S3. The Tribological Effects of ZrO_2-NPs in Cr Doped a-C Contacts

The tribological tests of ZrO_2-NPs dispersion in Cr-doped Please keep uniform a-C (H: 9.96 GPa) contacts were conducted to obtain the preliminary understanding of the influence of mechanical properties of a-C coating. The results in Figure S5 shows that ZrO_2-NPs dispersion could not reduce the friction, but significantly increased the wear rate for Cr-doped a-C, implying that the ZrO_2-NPs may be not suitable for the a-C coatings with the hardness below 10 GPa. Thus, the "polishing effect" between nanoparticles and a-C coatings should be depended on the diameter of nanoparticles, as well as the mechanical properties of a-C coatings.

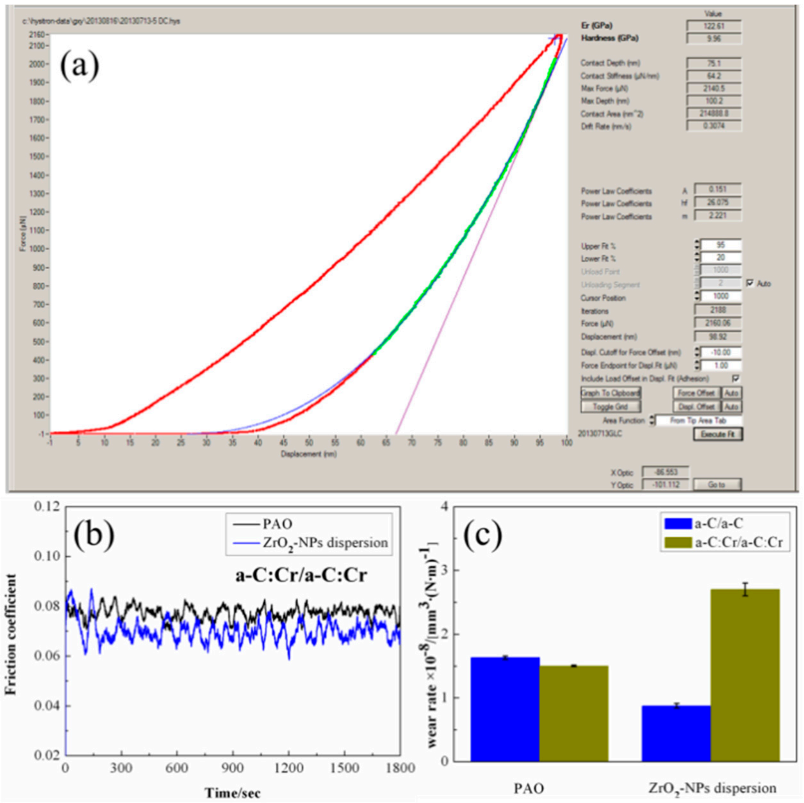

Figure S5. (**a**) The nano-indentation results of Cr doped a-C coating; (**b**) The COF of Cr doped a-C contacts under the lubrication of pure PAO and ZrO_2-NPs dispersion; (**c**) The wear rates of a-C and Cr-doped a-C under the lubrication of pure PAO and ZrO_2-NPs dispersion.

Section S4. The Stability of the ZrO_2-NPs Dispersions

Figure S6 presents the digital images of ultrasonically-dispersed ZrO_2-NPs dispersion in 12 h. It is seen that most of the ZrO_2-NPs could remain suspended in the PAO for 1 h, suggesting that the suspension was stable during the tribo-test (30 min). In addition, the oscillation of the disk may also prevent the sedimentation of ZrO_2-NPs. Nevertheless, modifying the surface of ZrO_2-NPs should have beneficial effects in preserving lubricating properties for long term use. The dispersants suitable for ZrO_2-NPs have been reported in the previous studies (*Monatsh. Chem.* **2008**, *139*, 183–195; *Surf. Rev. Lett.* **2007**, *14*, 1047–1052).

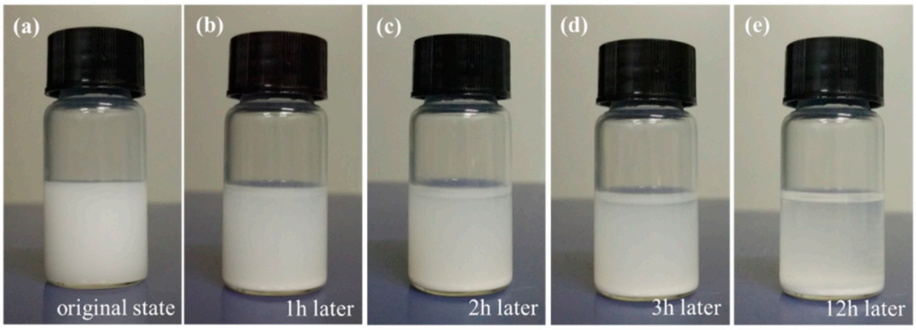

Figure S6. Digital images of dispersion of ZrO_2-NPs in PAO oil. The time for each picture is noted on the respective picture. Concentration of ZrO_2-NPs: 1 wt %. There is no explain of (**a**–**e**) in the caption, please add.
